# Supplementary material for: Genomic and Epidemiologic Surveillance of SARS-CoV-2 in the Pandemic Period: Sequencing Network of the Lazio Region, Italy
Source: Viruses. 2023 Oct 31;15(11):2192. doi: 10.3390/v15112192 (PMC10674723; doi:10.3390/v15112192)
Supplement: Supplementary file 1 [file viruses-15-02192-s001.zip › File S1.pdf]

Ion AmpliSeq SARS CoV 2 Insight Research Assay (ThermoFisher)

The assay enables research and surveillance of the SARS-CoV-2 virus, including variants of concern, through complete genome sequencing. It is based on Ampliseq designed primers sets released by ThermoFisher and is compatible with manual library preparation and sequencing on GeneStudio S5 Prime Sequencer, with automatic preparation by using DL8 kit on Chef instruments for Library prep and sequencing on GSS5 Prime Sequencer, and with Genexus Integrated sequencer for all-automated process for library preparation and sequencing.

COVIDSeq Assay (Illumina)

The assay leverages a modified version of ARTIC multiplex PCR protocol, with 99 amplicons designed to amplify SARS-CoV-2 virus-specific sequences, combined with proven Illumina sequencing technology. An optional primer pool is also available.

COVIDSeq Assay is compatible with Miseq, NovaSeq 6000, NextSeq 2000, or NextSeq 500/550/550Dx.

NEBNext® ARTIC SARS-CoV-2 Library Prep Kit for Illumina (New England Biolabs)

NEBNext ARTIC kits include primers and reagents for RT-PCR from SARS-CoV-2 gRNA and downstream library preparation for Illumina® and Oxford Nanopore Technologies® sequencing. The V3 ARTIC primers have been balanced, using methodology developed at NEB based on empirical data from sequencing. In combination with optimized reagents for RT-PCR, the kits deliver improved uniformity of amplicon yields from gRNA across a wide copy number range.
The NEBNext ARTIC SARS-CoV-2 Library Prep Kit generates libraries with inserts ~400 bp. If shorter inserts are preferred, the NEBNext ARTIC SARS-CoV-2 FS Library Prep Kit produces ~150 bp insert libraries compatible with 2 x 75 sequencing.

QIAseq DIRECT SARS-CoV-2 Kit A (QIAGEN)

4-hour workflow with no need for fragmentation or ligation reactions.

QIAseq DIRECT SARS-CoV-2 Kit is designed on short-read sequencing platforms. It generates amplicons that are approximately 250 bp, compared to 400 bp amplicons produced by alternative methods, resulting in shorter amplicons that don't require fragmentation.

QIAseq DIRECT SARS-CoV-2 libraries are compatible with Illumina NGS platforms including iSeq 100, MiniSeq, MiSeq, NextSeq 500/550, HiSeq 2500, HiSeq 3000/4000 and NovaSeq 6000.
