# Supplementary material for: Genomic and Epidemiologic Surveillance of SARS-CoV-2 in the Pandemic Period: Sequencing Network of the Lazio Region, Italy
Source: Viruses. 2023 Oct 31;15(11):2192. doi: 10.3390/v15112192 (PMC10674723; doi:10.3390/v15112192)
Supplement: Supplementary file 1 [file viruses-15-02192-s001.zip › File S2.pdf]

SUPPLEMENTAL TABLE

Data Availability

GISAID Identifier:	EPI_SET_230830rs
doi:	10.55876/gis8.230830rs

All genome sequences and associated metadata in this dataset are published in GISAID's EpiCoV database. To view the contributors of each individual sequence with details such as accession number, Virus name, Collection date, Originating Lab and Submitting Lab and the list of Authors, visit 10.55876/gis8.230830rs

Data Snapshot

- EPI_SET_230830rs is composed of 11,516 individual genome sequences.
- The collection dates range from 2020-01-29 to 2022-10-31;
- Data were collected in 1 countries and territories;
- All sequences in this dataset are compared relative to hCoV-19/Wuhan/WIV04/2019 (WIV04), the official reference sequence employed by GISAID (EPI_ISL_402124). Learn more at https://gisaid.org/WIV04.
