# Supplementary figures and images for: Genomic and Epidemiologic Surveillance of SARS-CoV-2 in the Pandemic Period: Sequencing Network of the Lazio Region, Italy
Source: Viruses. 2023 Oct 31;15(11):2192. doi: 10.3390/v15112192 (PMC10674723; doi:10.3390/v15112192)

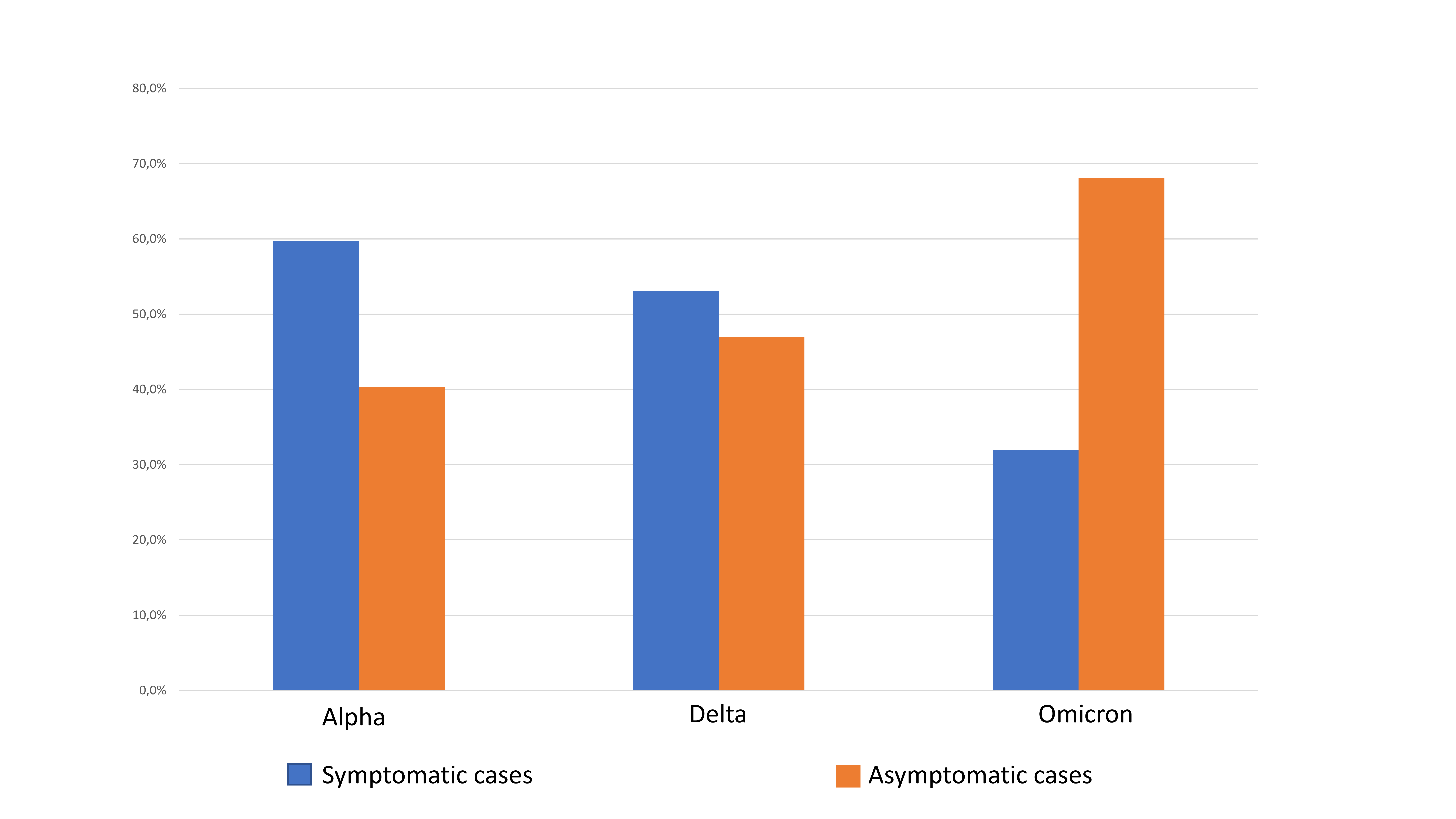

Supplement: Supplementary file 1 [file viruses-15-02192-s001.zip › Figure S1.jpg]

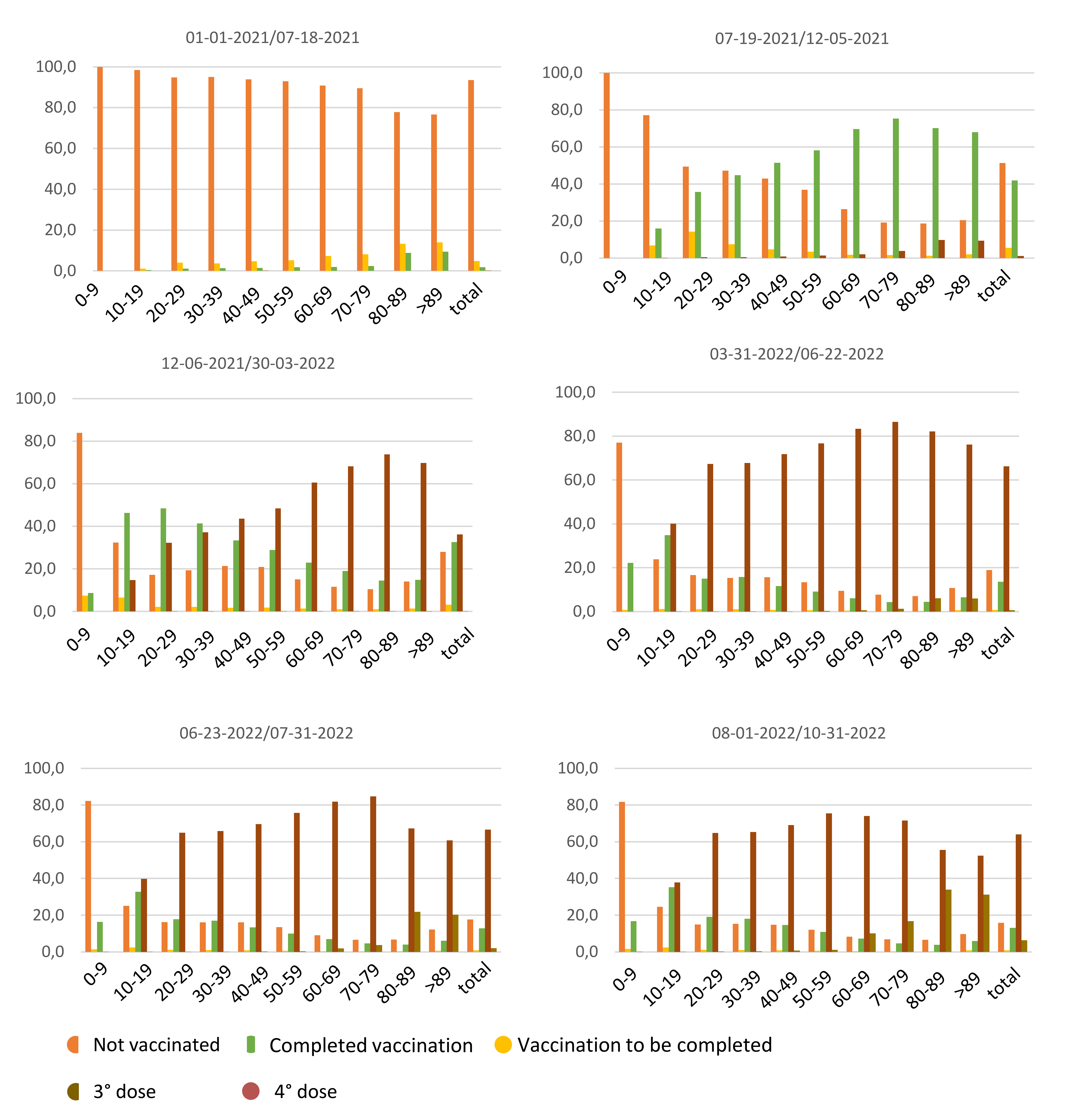

Supplement: Supplementary file 1 [file viruses-15-02192-s001.zip › Figure S2.jpg]
